# Assessment of patient satisfaction and preferences with an intensive care diary

**DOI:** 10.5935/0103-507X.20190028

**Published:** 2019

**Authors:** Teresa Tavares, João Camões, Daniela Rodrigues Carvalho, Rosa Jacinto, Cláudia Machado Vales, Ernestina Gomes

**Affiliations:** 1 Serviço de Medicina Interna, Hospital Pedro Hispano - Matosinhos, Portugal.; 2 Serviço de Medicina Intensiva, Hospital Pedro Hispano - Matosinhos, Portugal.

**Keywords:** Critical care, Deep sedation, Memory disorders, Patient satisfaction, Patient preference, Cuidados críticos, Sedação profunda, Transtornos da memória, Satisfação do paciente, Preferência do paciente

## Abstract

**Objectives:**

To evaluate the satisfaction of patients admitted to the intensive care unit using a diary and analyze possible points for improving this instrument.

**Methods:**

This was an observational, retrospective study, conducted between March 2014 and July 2017, in a multidisciplinary intensive care unit of a district hospital. The diary was implemented in patients sedated for 3 or more days. Three months after discharge, their satisfaction was assessed using a questionnaire. A patient who agreed with the 5 statements assessing the diary's help in clarifying the intensive care unit stay, in filling memory gaps, in recovery, in reassurance, and in the recommendation of this intervention was defined as satisfied.

**Results:**

A total of 110 patients were included, of whom 55 answered the questionnaire. Of these, 36 (65.5%) were classified as satisfied. Each item had a positive response in more than 74% of cases. A total of 60% of the participants suggested increasing the number of photographs. No significant differences were found in the subgroup analysis (age, sex, duration of sedation and ventilation, length of diary keeping, severity on admission, or delirium, depression, or anxiety in the intensive care unit).

**Conclusions:**

Most patients were satisfied with the diary but suggested an increase in the number of photographs.

## Abstract (pt)

**Objetivos:**

Avaliar a satisfação dos doentes internados em uma unidade de cuidado intensivo com o diário e analisar possíveis pontos de melhoria deste instrumento.

**Métodos:**

Estudo retrospectivo observacional, decorrido entre março de 2014 e julho de 2017, em uma unidade de cuidado intensivo polivalente de um hospital distrital. Foi iniciado o diário em doentes sedados por 3 ou mais dias. Depois de 3 meses da alta, foi avaliada a satisfação deles por meio de um questionário. O doente que concordou com as cinco afirmações que visavam avaliar esclarecimento, preenchimento de lacunas de memória, ajuda na recuperação, tranquilização e recomendação da intervenção foi definido como satisfeito.

**Resultados:**

Foram incluídos 110 doentes, dos quais 55 responderam o questionário. Destes, 36 (65,5%) foram classificados como satisfeitos. Cada item teve uma resposta positiva em mais de 74% dos casos. Sugeriram o aumento do número de fotografias 60% dos participantes. Não foram encontradas diferenças estatisticamente significativas na análise de subgrupos (idade, sexo, tempo de sedação e ventilação, tempo de diário, gravidade à admissão, *delirium*, depressão ou ansiedade na unidade de cuidado intensivo).

**Conclusões:**

A maioria dos doentes mostrou-se satisfeita com o diário, sugerindo, no entanto, o aumento do número de fotografias.

## INTRODUCTION

In recent years, there have been changes in the approach to patients admitted to the intensive care unit (ICU), namely, with regard to sedation and analgesia.^(1)^ The use of intensive care diaries dates back to the early 1980s in Denmark, during a time with longer periods of ventilation and sedation, a paradigm that has changed in recent years.^(2)^ Sedation withdrawal and mobilization as early as possible are increasingly advocated.^(3)^ This development requires the adoption of strategies for monitoring and preventing new problems to which these patients may be subjected. Although patients are less sedated, many still have little or distorted memory of their stay in the ICU, which is sometimes exacerbated by hallucinations and nightmares, with increased risk of developing posttraumatic stress disorder (PTSD), anxiety, and depression, which are part of post-intensive care syndrome (PICS).^(4,5)^

This lack of memory is emphasized by the fact that these patients have a strong need to know what happened during their hospitalization^(6)^ and need help to build a complete and coherent narrative of this phase of their lives.

The development of a diary in which photographs and facts, events, and emotions related to this time period are recorded by those accompanying the patient (health professionals and family members) has been identified as one of the strategies allowing the patient to assign meaning and coherence and to chronologically order the period of time when memories are absent or distorted.^(7)^ The presentation and content of diaries vary according to ICU and local/regional protocols, and they may even be included in an organized and integrated patient rehabilitation structure. Despite this heterogeneity, the content of the diary complies with established general rules.^(8,9)^ Questions remain as to the best way to apply the diary and the positive and negative implications for patients, family members, and health professionals.^(10-15)^ Despite the demonstrated benefits in reducing the incidence of PTSD, there is currently no evidence from randomized studies of the benefits and harms of implementing diaries in patients and their caregivers that allows recommending their widespread use as a therapeutic strategy.^(14)^ Some studies have evaluated patient satisfaction with diaries through questionnaires or informal interviews,^(16-19)^ but there are no data on the Portuguese population, with the exception of the contribution of different Portuguese ICUs to the multicenter study by Jones et al.^(7)^

The objectives of the present study were to evaluate patient satisfaction with the diary and to analyze possible points of diary improvement to optimize the use of this instrument.

## METHODS

This is a retrospective, observational, and descriptive study conducted at the Intensive Care Unit of *Hospital Pedro Hispano* (HPH), in a multidisciplinary ICU, which began implementing diaries in 2009.

At the HPH ICU, a diary is started for all patients older than 18 years who are sedated for 3 or more days. The diaries are kept by nurses and doctors and by relatives and friends of the patient. The writing is performed at least daily, and these individuals describe what the patient is going through, write messages of encouragement, and share feelings. The diary is kept until the date of discharge from the ICU, even after the sedation is discontinued. When the patient improves, they are given the diary. The patient is reassessed in person by the intensivist at the follow-up visit (FUV) 3 months after discharge, when the development of PICS is monitored and satisfaction with the diary is evaluated through a questionnaire. The exclusion criteria for applying this tool are illiterate patients or those with a severe psychiatric disorder, identification of problematic families (from the point of view of communication difficulties), lack of social support/coverage, and poor short-term vital prognosis.

Data on all patients with a diary started between March 2014 and July 2017 were collected. Demographic data, the presence of functional or cognitive limitations, hospital admission data (main diagnosis, severity score on admission, length of ICU stay, duration of sedation and invasive ventilation, and development of delirium and PICS during hospital stay) and FUV data (answers to the satisfaction questionnaire and PICS at 3 months) in the ICU's electronic database were analyzed. Regarding the presence of functional or cognitive limitations, no validated scale was used, and they were only defined as "present" or "absent" according to the clinical evaluation. The main diagnoses were coded according to the World Health Organization's (WHO) International Statistical Classification of Diseases and Related Health Problems (ICD-10) of 2016, with the necessary adaptations to the specific context. The severity score calculated on admission was the Simplified Acute Physiology Score (SAPS) II.^(20)^ Delirium was identified using the Confusion Assessment Method for the Intensive Care Unit (CAM-ICU)^(21)^ applied by ICU nurses or physicians. PICS was identified during hospital stay by the patient's care team and was divided into its physical dimensions (muscle weakness - measured by the manual muscle strength test - mobility disorders, and glottis dysfunction) and nonphysical dimensions (anxiety, depression, PTSD, and cognitive problems). PICS at 3 months was evaluated by the physician and nurse responsible for the FUV through clinical evaluation and application of the Hospital Anxiety and Depression Scale (HADS),^(22)^ using a cutoff of 10 points for each subscale, and of the Post-Traumatic Stress Syndrome 14-Questions Inventory (PSSS-14).^(23)^

Follow-up ended at the FUV (3 months after discharge) or before, in case the patient died or missed the visit. Patients who missed the FUV, who did not read the diary, and/or who did not answer the questionnaire were excluded from the study.

The satisfaction with the diary questionnaire was developed by the authors. It was then was reviewed by experts in the field of intensive care medicine and psychiatry and modified accordingly before its application. Before its application in the present study, the questionnaire was tested in 5 patients to assess their understanding of the questions, and changes were made in the language after this process. The questionnaire included 11 questions answered on a Likert scale and a space for suggestions for improvement. Five questionnaire items were formulated to assess patient satisfaction (clarification about what happened at the hospital, filling of memory gaps, help with recovery, reassurance, and recommendation of the intervention to individuals in similar situations) (Table 1). Patients who answered "I agree" or "I strongly agree" to these 5 questions were defined as satisfied. The set of questions used to assess satisfaction showed high internal reliability, with Cronbach's alpha of 0.75.

**Table 1:** Answers to the questions assessing satisfaction

	I strongly agree	I agree	Neutral	I disagree	Did not answer
n = 55					
I felt enlightened about what happened to me after reading the diary	29 (52.7)	19 (34.5)	4 (7.3)	1 (1.8)	2 (3.6)
The diary helped me fill some gaps in my memory	23 (41.8)	24 (43.6)	6 (10.9)	2 (3.6)	0
Reading the diary helped in my recovery	22 (40.0)	23 (41.8)	10 (18.2)	0	0
I felt calmer after reading the diary	22 (40.0)	19 (34.5)	11 (20.0)	1 (1.8)	1 (1.8)
I recommend making this diary for people in similar situations	39 (70.9)	14 (25.5)	1 (1.8)	0	1 (1.8)

Results expressed as n (%).

To evaluate possible improvements to the diary from the patient's point of view, the responses to closed questions on the number of photographs, quality and quantity of information, and time of diary delivery were analyzed, along with the response to an open-ended question in which the patients could express concerns and suggestions.

### Statistical analysis

The statistical software Statistical Package for the Social Sciences (SPSS) version 24 was used for statistical analysis. Continuous variables with normal distribution were analyzed by parametric methods and the remainder by nonparametric tests. For the categorical variables, the chi-square test was used.

To evaluate the relationship between some patient characteristics and hospital stay, several subgroups were defined a priori. Each of the variables age, duration of sedation, duration of ventilation, length of diary keeping, and SAPS II was categorized into 2 subgroups, considering the mean/median of each variable as the cutoff point. The variables delirium, depression, and anxiety in the ICU were categorized into 2 subgroups according to their presence or absence. Significance was set at p < 0.01.

The questionnaires were sent to all patients with the diary, accompanied by a letter explaining the study that emphasized that participation in filling out the questionnaire was voluntary and required authorization to use these data for the study in question. All data were treated anonymously and confidentially. The study was approved by the Ethics Committee of the HPH Knowledge Management Service of the Local Health Unit of Matosinhos.

## RESULTS

**Figure 1:**
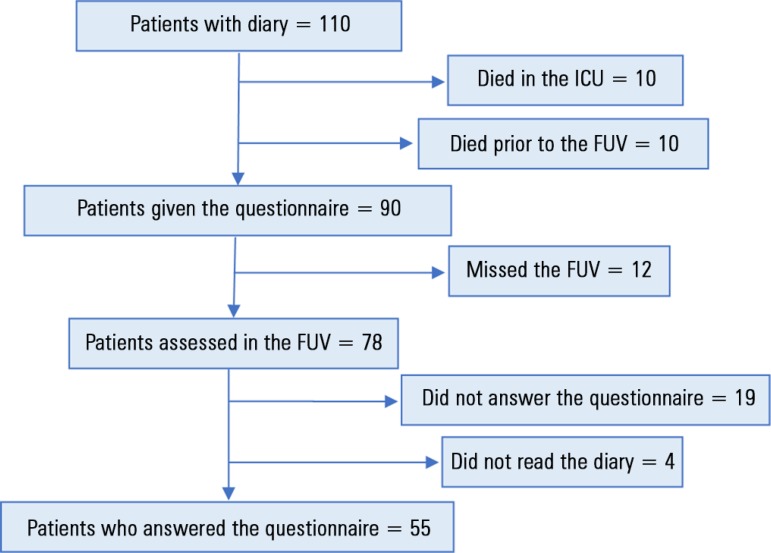
Flowchart of patients with diary and reasons for loss to follow-up. ICU - intensive care unit; FUV - follow-up visit.

During the study period, 1,300 patients were admitted to the ICU, and a diary was started for 110. As shown in figure 1, 20 patients died and 90 received the questionnaire. Twelve of these patients missed the FUV, 19 did not answer the questionnaire, and 4 did not read the diary, totaling 35 patients who were considered "nonparticipants". Thus, we evaluated the satisfaction of the remaining 55 patients.

Table 2 shows the demographic and clinical characteristics of the 90 patients who received the questionnaire. On average, the patients were on mechanical ventilation for 7 days and under sedation for 5 days and remained in the ICU for 11 days. A high percentage of patients developed delirium during the ICU stay (71.1%). The nonphysical components of PICS were more prevalent at 3 months than during hospitalization, and muscle weakness showed the opposite pattern. The most frequent cause of admission was sepsis (72.7%). The postoperative period of scheduled surgery was a reason for admission for only 2.7% of the patients with a diary. The median length of diary keeping was 9 days, with a minimum of 3 days and a maximum of 97 days. There were no significant differences between the participants and the nonparticipants (patients who missed the FUV and those who were evaluated in the FUV but did not answer the questionnaire or had not read the diary).

**Table 2:** Comparison between patients who did and did not answer the questionnaire.

	Participants (n = 55)	Nonparticipants (n = 35)	p value
Age	54.1 ± 16	59.1 ± 15	0.131
Male sex	39 (70.1)	21 (60.0)	0.768
SAPS II	41.6 ± 13	43.3 ± 16	0.577
Days of sedation	5 [3 - 40]	5 [1 - 36]	0.712
Days of invasive ventilation	7 [3 - 38]	7 [3 - 76]	0.699
Days in the ICU	11 [5 - 152]	11 [5 - 48]	0.581
Delirium in the ICU	38 (69.0)	26 (74.3)	0.596
Depression			
In the ICU	9 (16.3)	1 (2.9)	0.047
At 3 months	7 (12.7)	5 (21.7)*	0.314
Anxiety			
In the ICU	4 (6.0)	2 (5.7)	0.773
At 3 months	9 (16.4)	3 (13.0)*	0.711
PTSD			
In the ICU	0	0	1
At 3 months	3 (5.5)	2 (8.7)*	0.594
Muscle weakness			
In the ICU	41 (74.5)	26 (74.3)	0.978
At 3 months	5 (9.1)	3 (13.0)*	0.600

SAPS - Simplified Acute Physiology Score; ICU - intensive care unit; PTSD - post-traumatic stress disorder.

* In nonparticipants, the components of post-intensive care syndrome at 3 months were evaluated in 23 patients. Results expressed as the mean ± standard deviation, n (%) or median [CI].

Table 1 shows the answers to the questions assessing patient satisfaction with the diary. In each question, the percentage of patients who agreed with the statement was greater than 74%. Thirty-six patients (65.5%) agreed that the diary helped to clarify what had happened, to fill gaps in memory, to help in recovery, and to provide reassurance, so that they would recommend keeping it to people in similar situations and were thus classified as "satisfied".

Some participant characteristics that could influence the satisfaction with the diary were analyzed.

From the demographic point of view, the hypotheses that younger patients (median age defined as cutoff point, i.e., patients under 58 years of age) or female patients value this intervention more and were more satisfied than older or male patients were tested. No significant differences were found (Table 3).

**Table 3:** Analysis of satisfaction by subgroup.

	Satisfied (n = 36)	Not satisfied (n = 19)	p value
Age	54.2 ± 15.2	53.9 ± 17.0	0.506
Female sex	11 (30.6)	5 (26.3)	0.742
SAPS II	41.9 ± 14.2	41.0 ± 10.8	0.243
Days in the ICU	10 [5 - 152]	12 [5 - 48]	0.729
Days of ventilation	7 [3 - 58]	8 [1 - 37]	0.993
Days of sedation	4 [2 - 40]	4 [1 - 36]	0.505
Delirium	26 (72.2)	12 (63.2)	0.489
Depression			
In the ICU	5 (13.9)	4 (21.1)	0.703
At 3 months	5 (13.9)	2 (10.5)	1
Anxiety			
In the ICU	4 (11.1)	0	0.286
At 3 months	6 (16.7)	3 (15.8)	1
PTSD			
In the ICU	0	0	-
At 3 months	2 (5.6)	1 (5.3)	1
Muscle weakness			
In the ICU	26 (72.2)	15 (78.9)	0.749
At 3 months	4 (11.1)	1 (5.3)	0.649

SAPS - Simplified Acute Physiology Score; ICU - intensive care unit; PTSD - post-traumatic stress disorder. Results expressed as the mean ± standard deviation, n (%) or median [CI].

Based on the assumption that patients with greater potential to develop memory gaps would be those who would most benefit from reading the diary, the satisfaction of patients sedated (> 5 days) or ventilated (> 8 days) for longer, with longer length of diary keeping (> 9 days) and higher SAPS II (> 42), was compared with that of the remaining patients, and no significant differences were found among the various subgroups (Table 3).

Lastly, it was evaluated whether patients who developed psychological components of PICS (delirium, depression, or anxiety) in the ICU experienced greater or lesser benefit from the intervention, but this did not translate into satisfaction in a statistically significant manner (Table 3).

Possible points for improvement of the diary in the patient's perspective were evaluated. The responses are summarized in table 4. Among the patients, 60% thought that the diary should contain more photographs. Three patients reported problems in the clarity and amount of transmitted information. Six patients preferred that the diary had been delivered earlier.

**Table 4:** Answers to the questions that assess possible improvement points

	I strongly agree	I agree	Neutral	Disagreement	They did not answer
n = 55					
I think the diary should contain more photographs.	19 (34.5)	14 (25.5)	21 (38.2)	1 (1.8)	0
The information transmitted in the diary was clear and easy to understand.	22 (40.0)	27 (49.0)	3 (5.5)	3 (5.5)	0
The diary contained all of the information I needed to know about my hospitalization.	22 (40.0)	24 (43.6)	6 (10.9)	3 (5.5)	0
The diary should have been delivered earlier.	3 (5.5)	3 (5.5)	24 (43.6)	18 (32.7)	3 (5.4)
The diary should have been delivered to me later.	1 (1.8)	2 (3.6)	25 (45.5)	23 (41.8)	4 (7.3)

Results expressed as n (%).

## DISCUSSION

The main objective of the present study was to evaluate satisfaction with the diary by patients admitted to a portuguese ICU. Satisfaction was assessed by completing a questionnaire that addressed the items clarification, filling of memory gaps, help in recovery, reassurance, and recommendation of intervention in similar cases. Each of the items had a positive response in at least 74.5% of cases, and the item summarizing the patient's opinion reached a maximum of 96.4% (recommendation of the intervention in cases similar to yours). However, the authors were more restricted in the definition of satisfaction adopted, which considered only patients who answered positively to the 5 questions evaluating satisfaction, which occurred in 65.5% patients. Previous studies evaluating satisfaction have always used a qualitative method, and it is therefore not possible to compare these results with those of other cohorts.^(13,15,17,18,24)^

When the participants were subdivided according to demographic characteristics that could be associated with greater valuing of the diary (younger or female patients) or with indicators of greater severity (duration of sedation and ventilation, length of diary keeping, and severity score on admission), which could be associated with more memory changes and therefore with a greater benefit from the diary, there were no significant differences in the satisfaction of the different subgroups with the diary. Thus, we believe that it is beneficial to continue to start a diary for all these patients, regardless of age or the presence or absence of these severity indicators.

The same occurred with the development of psychological components of PICS, in which there was also no difference in the satisfaction of patients who did or did not develop *delirium*, depression, or anxiety in the ICU. Thus, we believe that including these patients in diary protocols remains essential.

There are no data in the literature on these subpopulations and on the specific application of the diary in these patients.

There are no specific and validated guidelines that define the structure, content, and process that the diary should present, and there is a diversity of diary-keeping practices among ICU.^(16)^ The inclusion of photographs of sedated patients who are unable to consent is among the most discussed aspects.^(11)^ The best time to deliver the diary to the patient is also controversial.^(17)^

To help answer these concerns, the objective of this study was to analyze possible points for improvement of this instrument, from the point of view of the patient.

Increasing the number of photographs was the most frequent suggestion, appearing in 33 of the 55 questionnaires. In the literature, photographs appear to be one of the aspects most appreciated by patients. Studies reveal that patients usually want more photographs and that these should depict times that are difficult to describe to individuals who did not visit them or less comfortable situations.^(16)^

Consistent with these data, 9 patients (16.4%) did not agree that the diary contained all the information they needed about their hospital stay. Some studies found that patients want detailed information, including on negative events, and the description of medical procedures and daily activities were some of the preferred contents.^(9,16,24)^ In a recent study, Teece et al. warned that reading the diary can arouse feelings of fear and anxiety, highlighting the importance of maintaining continued support, which can be provided through the FUV (as occurs in the HPH ICU) or support groups.^(11)^

For the narrative to be understandable, the patients included in the study by Åkerman et al. emphasized the importance of presenting the data in chronological order.^(16)^ In the present study, the main obstacle to information clarity was the difficulty in understanding the handwriting of some entries.

More than three-quarters of the patients agreed with the time of delivery of the diary. There is still no consensus as to the best time to do so. In some places, the diary is delivered during the hospital stay; in others, at the time of discharge; and sometimes, only later by mail.

There are some biases associated with the use of questionnaires, such as the social desirability bias (tendency to give an answer that is socially promoted/desirable) and the Hawthorne effect (participants behave differently when they know they are being observed), that we tried to avoid by using anonymous questionnaires, which could be completed at home and later turned in at the FUV. The use of a classic Likert scale with 5 responses would be more appropriate to evaluate patient satisfaction with the diary.

Two other biases to which this method is exposed are participation bias (individuals who agree to participate in the study are different from those who refuse and are not representative) and loss of follow-up (especially when a significant percentage of participants drop out of the study - 38.9% in this study). To evaluate its effect, we thoroughly characterized individuals who did not participate in the study or were lost to follow-up to determine how they differ from the participants. We concluded that in regard to the various analyzed parameters, these 2 groups do not present significant differences. Despite the lack of statistical significance (p = 0.314), the group of nonrespondents had a larger number of depressed patients (21.7% *versus* 12.7%), which may have influenced their adherence.

It is difficult to compare our results with others, given that the definition of "satisfaction with the diary" does not exist in the literature and was constructed by the authors. In addition, diaries are not uniform among ICU, especially with regard to the individuals writing them (inclusion or exclusion of family members), the instructions given to them, and the inclusion or not of clinical information. In this sense, there could be advantages in developing more specific recommendations regarding the method of application of this instrument. However, some authors believe that "very strict guidelines" could negatively affect or inhibit the writing and sharing of feelings by family members and health professionals.^(25)^

More studies are needed, especially multicenter prospective studies, given the importance of cultural differences among countries. Only then will it be possible to improve this instrument and develop recommendations that assist in its use in patients admitted to the ICU and possibly extend its use to other groups of patients, inside and outside the ICU.

## CONCLUSION

The data from this study suggest that 65.5% patients were completely satisfied with the diary. There were no differences in satisfaction when analyzing different patient characteristics, such as age, sex, presence of severity indicators, or development of delirium, depression, or anxiety in the intensive care unit. Based on this study and the published literature, the authors recommend implementing diaries in all patients admitted to the intensive care unit and sedated for 3 or more days.
